# Identification, Genome Sequencing, and Characterizations of *Helicobacter pylori* Sourced from Pakistan

**DOI:** 10.3390/microorganisms11112658

**Published:** 2023-10-29

**Authors:** Anees Fatima, Muhammad Ibrahim, Adil Naseer, Arshid Pervez, Muhammad Asad, Aamer Ali Shah, Fariha Hasan, Wadi B. Alonazi, Ifra Ferheen, Samiullah Khan

**Affiliations:** 1 Department of Microbiology, Faculty of Biological Sciences, Quaid-I-Azam University, Islamabad 45320, Pakistan; aneesfatima@aust.edu.pk (A.F.); alishah@qau.edu.pk (A.A.S.); farihahasan@yahoo.com (F.H.); 2 Department of Microbiology, Faculty of Health & Biological Sciences, Abbottabad University of Science & Technology, Abbottabad 22500, Pakistan; 3 Department of Biosciences, COMSATS University Islamabad (CUI), Sahiwal Campus, Sahiwal 55000, Pakistanmasadhiraj@gmail.com (M.A.); 4 Department of Gastroenterology, Ayub Medical College, Main Mansehra Road, Abbottabad 22020, Pakistan; adilalizai@gmail.com; 5 Department of Environmental Sciences, COMSATS University Islamabad (CUI), Abbottabad Campus, University Road, Tobe Camp, Abbottabad 22010, Pakistan; pervez@cuiatd.edu.pk; 6 Health Administration Department, College of Business Administration, King Saud University, Riyadh 11587, Saudi Arabia; waalonazi@ksu.edu.sa; 7 School of Biosciences and Veterinary Medicine, University of Camerino, 62032 Camerino, Italy; ifra.ferheen@unicam.it

**Keywords:** *H. pylori*, genome, drug resistance, virulence, comparative analysis, epidemiology

## Abstract

The stomach’s colonization by *Helicobacter pylori* (*H. pylori*) results in gastritis, ulcers, and stomach cancer. Frequently, pain is treated with medication, but resistant *H. pylori* infections are not. Therefore, it is important to find pharmacological targets and improved treatments for resistant *H. pylori* strains. The aim of the current study was sampling, identification, drug susceptibility testing following genome sequencing and comparative genome-wide analysis of selected *H. pylori* strains from Pakistan with three representative strains for virulence and drug-resistant characteristics. Based on culture, biochemistry, and molecular biology, 84 strains of *H. pylori* were identified, which made up 47% of the enrolled cases. Among all *H. pylori* strains, the highest resistance was reported for metronidazole with 82 *H. pylori* strains (98%), followed by clarithromycin with 62 resistant strains (74%). Among metronidazole-resistant strains, 38 strains (46%) were also resistant to clarithromycin, contributing 61% of clarithromycin resistant cases. Two strains, HPA1 and HPA2, isolated from ‘gastritis’ and ‘gastric ulcer’ patients, respectively, were further processed for WGS. The draft genome sequences of *H. pylori* strains HPA1 and HPA2 encode 1.66 Mbp and 1.67 Mbp genome size, 24 and 4 contiguous DNA sequences, and 1650 and 1625 coding sequences, respectively. Both the genomes showed greater than 90% similarity with the reference strain *H. pylori* ATCC 43504/PMSS1. The antibiotic-resistant genes were identified among all the strains with overall similarity above 95%, with minor differences in the sequence similarity. Using the virulent gene data obtained from the Virulence Factor Database, 75 to 85 virulent genes were identified in the five genome assemblies with various key genes such as cytolethal distending toxin (*cdt*), type IV secretion system, *cag* PAI, plasticity region, cell-motility- and flagellar-associated genes, neutrophil-activating protein (HP-NAP), T4SS effector cytotoxin-associated gene A (*cagA*), and urease-associated genes *ureA* and *ureB,* etc. Sequence similarity between the virulence factors found in this study and reference genes was at least 90%. In summary, the results of our study showed the relationship between clinical results and specific *H. pylori* strains’ (HPA1 and HPA2) genetics such as antibiotic resistance and specific virulence factors. These findings provide valued understanding of the epidemiology of *H. pylori*-associated diseases. Moreover, identification and genomics analysis have provided insights into the epidemiology, genetic diversity, pathogenicity, and potential drug resistance genes of *H. pylori* strains, offering a foundation for developing more targeted and effective medical interventions, including anti-virulent medications.

## 1. Introduction

The incidence of *H. pylori* infection differs across different regions and populations, but according to an estimate, more than 50% of the global population may be infected with this bacterium. It is particularly prevalent in underdeveloped nations such as Pakistan, where over 80% of the population carries *H. pylori* infection [1]. *H. pylori* infection is related to a range of gastrointestinal and systemic conditions such as peptic ulcers, iron deficiency, gastric mucosa-associated lymphoid tissue lymphoma, idiopathic thrombocytopenic purpura, and dyspepsia, and is primarily transmitted through the fecal–oral route [2,3]. In 1994, *H. pylori* was identified as a class-1 carcinogen; since then, it has been associated with stomach cancer, which is the second main cause of cancer-related deaths across the globe [3,4].

Antimicrobial combinations, such as metronidazole, clarithromycin, doxycycline, azithromycin, amoxicillin, and levofloxacin, coupled with bismuth salts and a proton pump inhibitor, are used to deal with *H. pylori* infection for a duration of 7–14 days or even longer. The main factor in treatment failures for *H. pylori* infections, along with the emergence of resistance to antibiotics, is patient adherence to medication [5]. The possibility of reinfection further complicates matters, particularly in regions where *H. pylori* infection rates are high [6].

Healthcare systems all over the world including Pakistan have been financially burdened by the management of *H. pylori* infections. Because of the growth of antibiotic resistance, it is getting harder. A 2018 meta-analysis systematic investigation found that 15% of approximately 66,000 isolated strains of *H. pylori* were resistant to clarithromycin, metronidazole, and levofloxacin [7,8]. The widespread resistant bacteria, with the burden they pose on community healthcare system, highlight the notion of drug repurposing as an approach to find novel uses for pre-existing medications [9]. This technique can save money, time, and energy because pharmacokinetics qualities and the safety of statins, anti-inflammatory, anthelmintic, anti-psychotic, and anti-cancer medicines have been demonstrated to have anti-bacterial potential [10].

It is also recognized that several virulence factors (VFs) affect how harmful *H. pylori* is. Presence of the VFs, e.g., blood group antigen-binding adhesin, the duodenal ulcer-promoting gene A, the vacuolating cytotoxin, the cytotoxin-associated gene pathogenicity island (*cag* PAI), and contact with epithelium are attributed to the presence of their respective genes, i.e., gene *babA*, *dupA*, *vacA*, *cag* PAI, and *iceA*, respectively, in the *H. pylori* genome. Varied genotypes of virulence genes are associated with different disease outcomes, and geographic distributional differences have also been documented [11]. This may also have an impact on how an infection turns out, especially if more serious side effects like stomach cancer develop. Moreover, various VFs are related to specific pathological conditions. Invasiveness of pathological consequences and severity of the symptoms depends upon the type and expression of strain-specific VFs [12]. In addition, new methods for discovering antimicrobial drugs are now being developed [13]. These methods are based on metabolic pathway analysis, genomics and proteomics, reverse docking, and essentiality of gene analysis [14]. They primarily rely on analysis of a pathogen’s genomic and proteomic sequencing data, using a variety of bioinformatic tools through different subtractive approaches, such as resemblance to the human proteome, predicted sub-cellular destination, and vitality towards the pathogen [15].

A comprehensive investigation of the complete genetic diversity of *H. pylori* strains provides crucial information in understanding its VFs, drug resistance, and single-nucleotide polymorphism (SNP) [16,17,18]. Further, presence of specific VFs indicates likelihood of severe disease progression. In order to design risk assessment studies, epidemiological information of the region and ethnicity could be useful [19,20]. *H. pylori* incidence is considerably high in South Asian populations. Such genome-based studies have compressively reported virulent and drug-resistant strains of *H. pylori* from Bangladesh, India, and China [21,22]. However, to the best of our knowledge, no comprehensive genome-based studies have been reported of *H. pylori* from Pakistan. This study investigates identification, drug susceptibility testing, genome sequencing, and computational genome-wide analysis for virulence and drug-resistant characteristics of *H. pylori* strains from Pakistan, with comparisons to three representative strains. It will be helpful to better understand the nature of local strains from Pakistan and their possible exploitation in *H. pylori* management [23,24]. Understanding these factors is crucial for developing strategies to manage and treat *H. pylori*-related diseases, particularly in the context of antibiotic resistance and virulence. This work contributes to the broader field of infectious disease research and the development of targeted therapies.

## 2. Materials and Methods

### 2.1. Isolation of H. pylori

Ethical permission for conducting this research study was approved by the research ethics committee of the Medical Teaching Institute, Abbottabad. A total of 177 patients participated in this research. The study was clarified to each participant, and informed written permission was also obtained from each participant.

**Figure 1:**
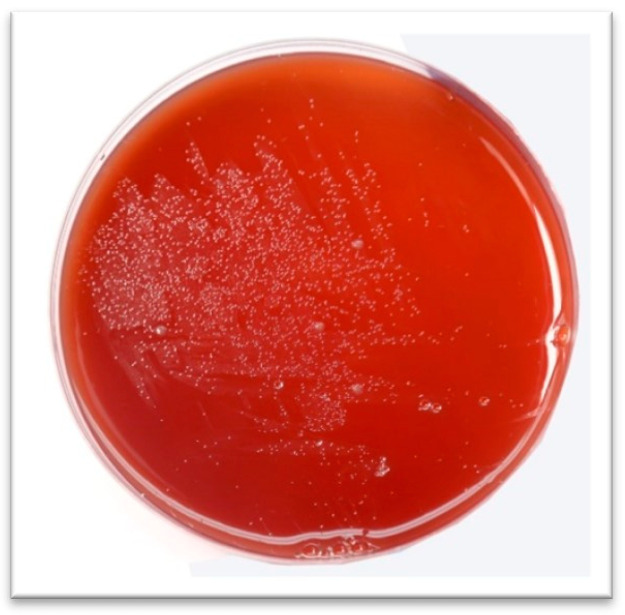
*H. pylori* growth on Columbia agar base, complemented with laked horse blood and Dent supplement.

Antral gastric biopsy samples were collected in 20% sterile glucose from patients attending endoscopy unit at the gastroenterology department, Ayub teaching hospital, Abbottabad, for upper gastrointestinal endoscopy. The collected biopsies were immediately transferred to the microbiology research lab, Comsats University Islamabad, Abbottabad campus for further processing. These biopsy samples were homogenized by using a tissue homogenizer and inoculated on freshly prepared Columbia blood agar (7% laked horse blood, SR 0048, Oxoid) complemented with *H. pylori* discriminating Dent’s supplement (SR0147E Oxoid) comprising trimethoprim (2.5 mg/2 mL), vancomycin (5 mg/2 mL), amphotericin B (2.5 mg/2 mL), and cefsulodin (2.5 mg/2 mL). These inoculated plates were incubated under moist and microaerophilic conditions at 37 °C for 7 days. A microaerophilic environment (5% O_2_, 10% CO_2_, and 85% N_2_) was provided by a microaerophilic gas-generating kit system (Campygen CN0025A, Oxoid UK) in 2.5 L jar (OxoidTM, AneroJarTM 2.5 L). The plates were observed regularly after three days for any growth. Small, round, pinpointed, translucent colonies were assumed to be *H. pylori*. These colonies were picked and sub-cultured twice for pure cultures (Figure 1) under conditions as discussed. Based on Gram-negative small s-shaped rods positive for urease, oxidase and catalase were presumed as *H. pylori* [25].

### 2.2. Molecular Confirmation of H. pylori via 16S RNA and Antibiotic Susceptibility Testing

**Figure 2:**
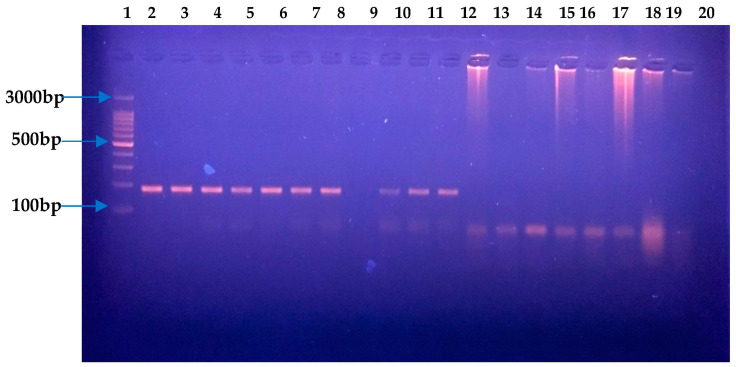
PCR amplification of genus-specific 16S for *H. pylori.* Well 1 contains 100 bps ladder, well 2 contains positive control, and lane 14 contains negative control. Lanes 3–8, 10–13, and 15–20 contain *H. pylori*-specific PCR products of bacterial strains isolated from gastric biopsy samples.

**Figure 3:**
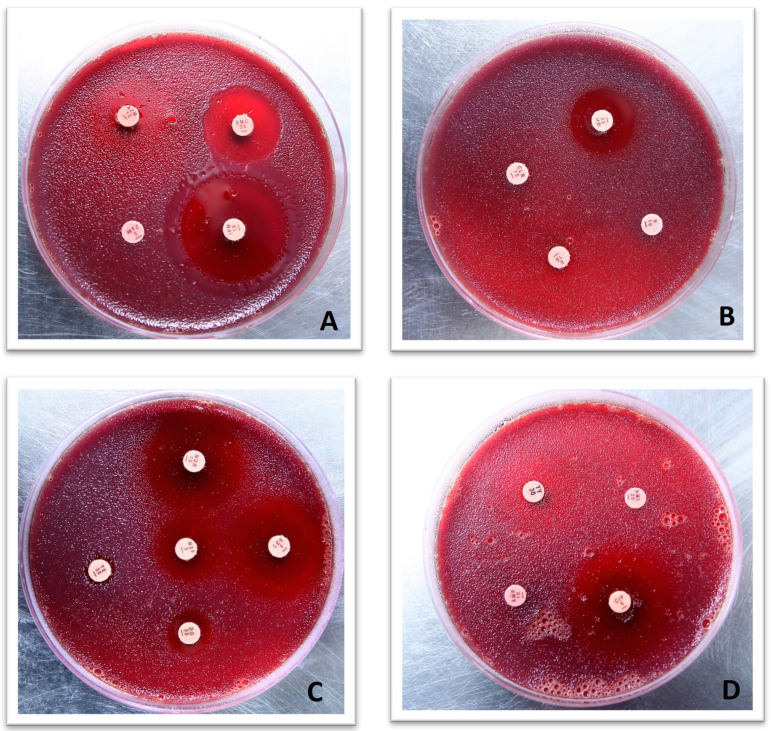
Antibiotic resistance profile of different antibiotics on *H. pylori* strain HPA2. (**A**). Antibiotic sensitivity testing against imipenem (IPM), amoxicillin/clavulanic acid (AMC), metronidazole (MTZ), and azithromycin (AZM). (**B**). Antibiotic sensitivity testing against clarithromycin (CLR), penicillin G (P), erythromycin (E), and minocycline (MH). (**C**). Antibiotic sensitivity testing against norfloxacin (NOR), tetracycline (TE), moxifloxacin (MXF), doxycycline (DO), and levofloxacin (LEV). (**D**). Antibiotic sensitivity testing against ciprofloxacin (CIP), ampicillin (AMP), and amoxicillin (AML).

Based on biochemical, Gram staining, and microscopic examination, strains initially screened as *H. pylori* strains were confirmed as such through PCR by using *H. pylori*-specific primers for 16S RNA as reported earlier [26]. A DNA isolation kit (GeneJET Genomic DNA purification kit #K0721, (Thermo Scientific, Waltham, United State)) was used to extract genomic DNA as per the manufacturer’s instructions. The primer sequence was HPF 5′-GCG ACC TGC TGG AAC ATT AC-3′ and HPR 5′-CGT TAG CTG CAT TAC TGG AGA-3′. *H. pylori* (NCTC 13282) was used as a positive control. Initial denaturation was performed for 5 min at 95 °C. Thirty-five cycles to amplify the target 16S RNA sequence were carried out. Each PCR cycle consisted of 30 s at 94 °C, 30 s at 55 °C, and 30 s at 72 °C. Lastly, elongation was achieved for 10 min at 72 °C. Gel electrophoresis was used to visualize amplified product. Next, 1.5% agarose gel in TBE containing ethidium bromide, loaded with PCR product, was subjected to electrophoresis for 45 min at 100 V. The amplified product among *H. pylori*-positive samples was 138 bps (Figure 2). These strains were further checked for their antibiotic sensitivity potential. Antibiotic susceptibility testing (AST) towards selective antibiotics was assessed by the disk diffusion method. Metronidazole, clarithromycin, amoxicillin, ampicillin, levofloxacin, and tetracycline were assessed for sensitivity. Mueller Hinton agar (Oxoid, CM0337) containing 10% horse blood was seeded with a cell suspension calibrated at 3 McFarland units for AST. Selected antibiotic disks were placed on media seeded with bacterial culture, and these plates were placed under a moist and microaerophilic environment at 37 °C. AST results (Figure 3) were observed after three days of incubation. Based on hetero-resistance, one strain isolated from a gastritis patient and one from a gastric ulcer patient were selected for further whole-genome sequence (WGS) analysis.

**Figure 4:**
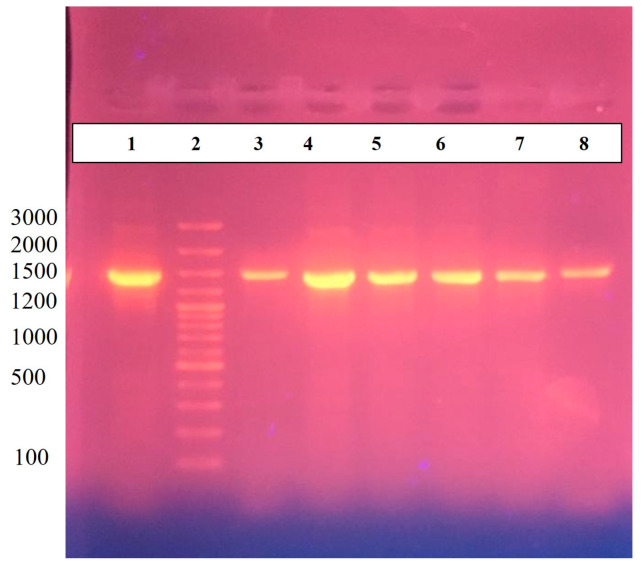
PCR amplification of 16S for RNA gene of confirmed *H. pylori* strains, HPA1 and HPA2. Well 1 contains positive control, well 2 contains 100 bps ladder, and lanes 3–8 contain HPA1, HPA2, HPA3, HPA4, HPA5, and HPA6, respectively.

Before processing for WGS, further confirmation was performed through *H. pylori* 16S RNA gene sequence. In addition to *H. pylori* strains HPA1 and HPA2, four more strains, HPA3, HPA4, HPA5, and HPA6, were also processed for PCR using universal primers for bacterial 16S rRNA, i.e., 27F and 1495R for amplification of the target DNA, as previously described [27]. The primer sequence for 16S universal forward primer (27F) was 5′-AGA GTT TGA TCC TGG CTC AG-3′, and 16S universal reverse primer (1495R) was 5′-CTA CGG CTA CCT TGT TAC GA-3′. PCR was carried out with initial denaturation at 95 °C for 5 min. For amplification, 30 cycles, each consisting of 94 °C for 1 m, 58 °C for 1 m, and 72 °C for 2 min were conducted. Final elongation was achieved at 72 °C for 10 min. Amplified product was seen through gel electrophoresis. Next, 1.5% agarose gel in TBE was subjected to electrophoresis for 1 h at 100 V. An approximately 1500 bp long fragment of DNA was achieved (Figure 4). The amplified product was sequenced through ‘*Macrogen*’ South Korea. The sequences of 16S RNA of *H. pylori* HPA1 and *H. pylori* HPA2 were submitted to NCBI under accession numbers OR262895 and OR535252, respectively.

**Figure 5:**
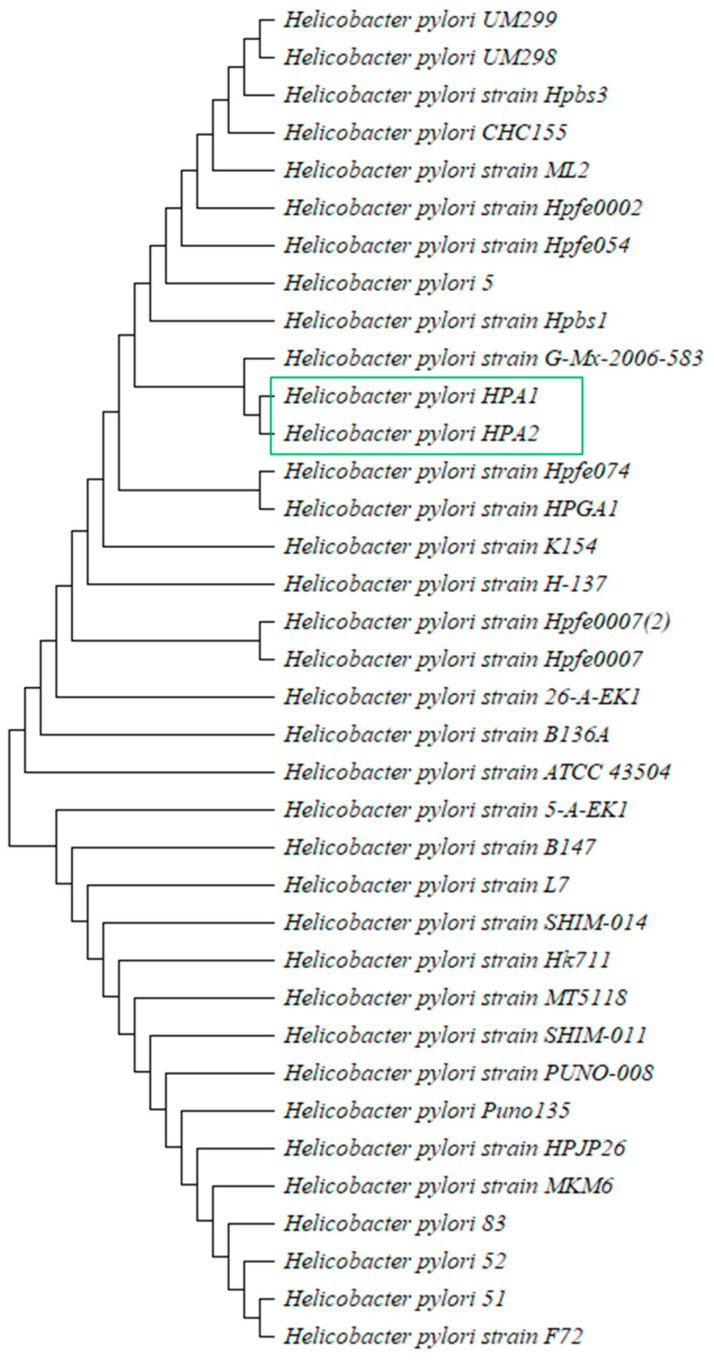
Genetic diversity of *Helicobacter pylori* strain HPA1 and strain HPA2.

Using the basic local alignment search tool for nucleotide (BLASTn) services, the 16S RNA sequence further confirmed that it was *H. pylori.* A phylogenetic tree was constructed to find out the genetic origins of both strains, i.e., HPA1 and HPA2 (Figure 5). Purified DNA of both strains was further processed for WGS using commercial services by ‘*Macrogen*’ South Korea.

### 2.3. Library Preparation, Genome Sequencing, and Assembly

As per the manufacturer’s instructions, gDNA was extracted from the confirmed samples, following construction of the library using the Nextera XT Kit (Illumina) for WGS. A quantity of 1 ng of DNA was amplified by using limited-cycle PCR (12 cycles) with Nextera XT barcodes.

Briefly, a MiSeq Illumina (‘*Macrogen*’ South Korea) was used for WGS. With the use of cut adapt 1.4, final sequences, the Illumina adaptors, bases beneath Q20, and unidentified bases were all eliminated from FastQ files during the refinement procedure. Using the Rqc v1.10.2 R package, the Q score and fractions of the A, C, G, and T variables were calculated. Quality-filtered reads from the *H. pylori* strains HPA1 and HPA2 were aligned using Bowtie v2.3.3.1. The reads were arranged by chromosomal location using Picard Tools v2.15.0. When readings overlapped, FLASH2 v2.2.00.21 was employed to combine them in a single read. Genius Prime v3 de novo sequencing assembly was used to confirm the discovery of entire genes missing from the formerly discovered *H. pylori* strain. Annotation was performed by rapid annotation using subsystem technology RAST for subsystem analysis based on cluster of orthologues.

### 2.4. Comparative Genomes Analysis

The genome comparison of *H. pylori* strain HPA1, *H. pylori* strain HPA2, *H. pylori* strain HPGA1, and *H. pylori* strain hpfe074 with representative gastric-disease-associated strains such as *H. pylori* strain American type culture collection 43504 (ATCC 43504) was carried out by using the BRIG software (0.95) package [28]. The BRIG software package is a bioinformatics tool used for visualizing and comparing multiple genomes following multiple genomes’ alignment and analysis of evolutionary processes such as inversion, rearrangement, similar subsequences, and comparison. Furthermore, guanine and cytosine content (GC content) and GC skew map were also drawn to visualize the drug-resistant gene distributions in strains HPA1 and HPA2 by using circular genome View (CGView) Server V1.0 [29].

### 2.5. Genome-Wide Drug-Resistant and Virulent Gene Analysis

We acquired the FASTA genetic sequences from the virulence factor database (VFDB) to identify the virulence genes for the *H. pylori* strains HPA1, HPA2, HPGA1, hpfe074, and ATCC 43504 genomes. Across more than 30 different bacterial genera, this version of the VFDB had more than 30,178 genes that encoded more than 1800 distinct VFs. All virulent genes set were analyzed with the genomes of the *H. pylori* strains HPA1, HPA2, HPGA1, hpfe074, and ATCC 43504. This was performed using the basic local alignment search tool (BLAST) v2.7.1. Only sequences which covered 80% or higher similarity when the VFDB query gene was used were retained. Moreover, virulence genes’ loci were also retrieved by using the VFDB-based identified gene sequences to the RAST annotations for each of the genomes examined here as reported by Paniagua-Contreras et al. [30], as noted in Table S2. In the VFDB, 349 genes were found to have orthologous genes. These 349 genes were manually classified into several functional groups [31,32,33].

## 3. Results

### 3.1. H. pylori Culture and Antimicrobial Susceptibility Testing

A total of 177 samples were processed. Based on culture, biochemistry, microbiology, and *H. pylori*-specific polymerase chain reaction (PCR), 84 strains were identified, which made up 47% of the enrolled cases. The pure culture of *H. pylori* appeared as sparkling translucent colonies (Figure 1) when incorporated on Columbia supplemented agar. Metronidazole, clarithromycin, amoxicillin, ampicillin, levofloxacin, and tetracycline were assessed for resistance.

Based on phenotypic resistance towards antibiotic discs, *H. pylori* isolates were considered resistant. All 84 strains were sensitive to ampicillin, 80 strains (95%) were sensitive to amoxicillin, 82 strains (98%) were sensitive to levofloxacin, and 81 strains (96%) were tetracycline-sensitive. Among all *H. pylori* strains, only 02 strains (02%) were sensitive to metronidazole, and 22 of the strains (26%) were sensitive to clarithromycin (Table 1).

Among all *H. pylori* strains, the highest resistance was reported for metronidazole with 82 *H. pylori* strains (98%), followed by clarithromycin with 62 resistant strains (74%). Among metronidazole-resistant strains, 38 (46%) were also resistant towards clarithromycin, contributing 61% of clarithromycin-resistant cases. This co-resistance is largely responsible for treatment failure and infection persistence in communities.

### 3.2. Identification of H. pylori and Evolutionary Analysis

In the current study, isolation of *H. pylori* was successfully carried out from the gastric biopsies of gastritis and ulcer patients. Gram-negative and urease-, catalase-, and oxidase-positive isolates with a distinguishing morphology under the microscope were recognized as *H. pylori*. Out of the 84 DNA extracts from pure cultures, part of the 16S rRNA gene specific to *H. pylori* was amplified through PCR.

Sequence analysis of partial 16S rRNA gene of HPA1 and HPA2 obtained through PCR using universal primers confirmed *H. pylori* by BLAST displaying 99–100% similarity with *H. pylori* 16S rRNA sequences found in GenBank databases. Phylogenetic investigation and a cladogram (Figure 5) showed that strains under study are close to *H. pylori* strain HPGA1 and Hpfe074 and have the same cluster.

### 3.3. Genomic Features of H. pylori Strains

Following overall genomic characteristics of *H. pylori* strains revealed the various key genomic features. Draft genome sequences of *H. pylori* strains HPA1 and HPA2 isolated in this study from gastritis and gastric ulcer patients are of 1.66 Mbp and 1.67 Mbp and contained 24 and 4 contigs of DNA sequences, respectively. The genome of *H. pylori* strain HPA1 encodes 1,650 coding genes, 33 tRNA, 10 rRNA, and has 38.8% GC content. Similarly, the genome of *H. pylori* strain HPA2 encodes 1625 coding genes, 35 tRNA, 9 rRNA, and has 38.7% GC content. The *H. pylori* strain HPGA1 has a genome size of 1.50 Mbp and encodes 1596 coding genes, 36 tRNA, 4 rRNA, 39.9% GC content with a single chromosome. The genome sequence of *H. pylori* strain hpfe074, a causative agent of infections in gastroduodenal tract of humans, comprised 1.67 Mbp long single circular chromosomal DNA, along with a plasmid. Its genome has a GC content of 38.71%. Furthermore, it encodes 1629 genes, 4 rRNAs, and 35 tRNAs (Table 2). The reference genome sequence of *H. pylori* strain, ATCC 43504, used in this study, possesses two contagious DNA sequences collectively of 1.6 Mbp (Table 2).

### 3.4. Comparative Genome Analysis

The sequence-based comparison results were obtained for relative comprehensive genome investigation, for *H. pylori* strain HPA1, *H. pylori* strain HPA2, *H. pylori* strain hpfe074, *H. pylori* strain HPGA1, and *H. pylori* reference strain ATCC 43504. The BLAST ring image generator (BRIG) software [34] and RAST server analytical tools were used for this study.

**Figure 6:**
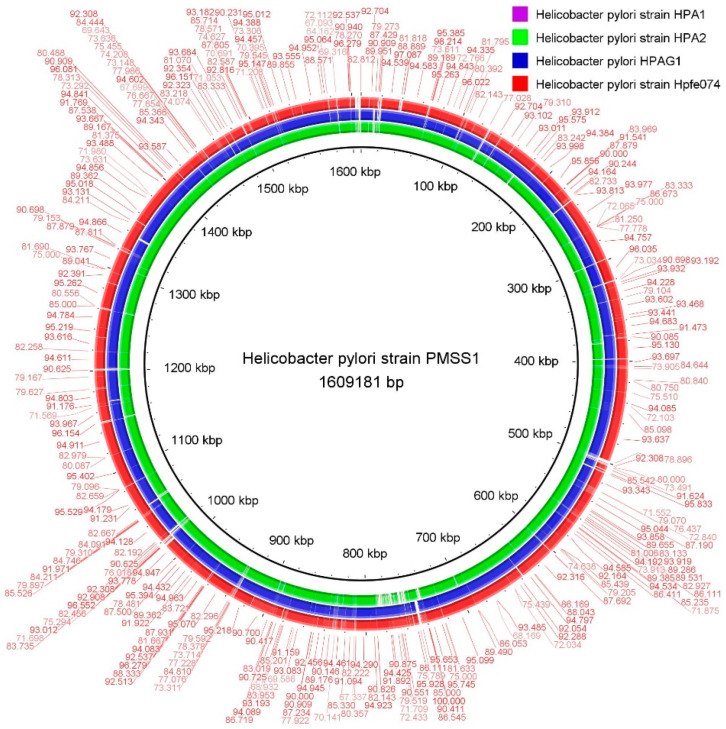
Comparative genome-wide analysis of *H. pylori* strains HPA1, HPA2, and reference strains.

The *H. pylori* reference strain ATCC 43504 was used to visualize the genomic areas (Figure 6). The circular genomes of the *H. pylori* strain HPA1, *H. pylori* strain HPA2, *H. pylori* strain HPGA1, *H. pylori* strain hpfe074, and *H. pylori* strain ATCC 43504 revealed the coding sequence (CDS), the percentage age of GC contents, the total number of RNAs, and the open reading frame (Figure 6). The visual examination of the whole genomes showed substantial similarity levels (>98%) with many positions of dormant deletion/insertion depicted sites among the genomes of *H. pylori* strains under study, i.e., HPA1, HPA2, HPGA1, hpfe074, and ATCC 43504. While similarity with remaining genomes ranged between 90% and 95%. Furthermore, these strains coded numerous proposed genes consistent with previous genome-wide studies.

### 3.5. Identification and Characterizations of Antimicrobial Resistance Genes

A ‘resistant gene identifier’ anticipated the presence of genes related to antibiotic-resistance in genomics assemblies of all five strains and found that various antibiotic-resistant genes were found distributed among all five strains, indicating a wide distribution of antibiotic resistance among these strains. Overall, the antibiotic-resistant genes were identified among all the strains except some differences in the similarity such as ranging from 95% to 99% in *H. pylori* strains HPA1 and HPA2 when compared with the reference strain *H. pylori* ATCC 43504. The key drug-resistant genes identified in our study were DNA gyrase subunit A (*gyrA*), Enoyl-[acyl-carrier-protein] reductase, *cme*DEF [multidrug efflux system], Uridine Diphosphate-N-acetylglucosamine (UDP-N-acetylglucosamine) 1-carboxyvinyltransferase, and transcription termination factor ‘*rho*’ in sequences of strains HPA1 and HPA2 with the reference strain, while in *H. pylori* hpfe074, the similarity was noted (Table S1). Moreover, the CG view circular image visualized by the CGView tool confirmed the location of the drug-resistant genes, mutations, and distribution as shown in Figures S1 and S2 with GC contents and rRNA, tRNA, and coding sequences. The results showed strains HPA1 and HPA2 to be the potential drug-resistant strains identified in this study.

### 3.6. Detection of Virulence Genes and Analysis

Comprehensive dataset of virulence genes was retrieved from the VFDB. Various keywords such as acid resistance, adherence, immune evasion, immune modulator, motility, secretion system, and toxin were used to proceed for BLAST analysis for each genome assembly. A total of 65 to 85 virulence factor genes were found among all five genome assemblies. *H. pylori* strains HPA1 and HPA2 encode 68 and 69 key virulent genes, *H. pylori* strain HPGA1 encodes 71, and *H. pylori* strain hpfe074 comprised 72 virulent genes, dispersed at several genomics areas. Among these virulence-related genes, 66 were common to all five *H. pylori* strains. Among these common genes, mostly genes involved in iron uptake, including iron/manganese transport genes such as *horB*, *hes*, *fepG*, *entB*, *entE*, *Ferric enterobactin transport protein FepE*, *salicylate synthase Irp9, enterobactin exporter* genes, and adherence- and motility-associated genes, were included.

In brief, the VFs recognized in this study comprised characteristics from both databases with at least 90% similar sequences with reference genes. Generally, *H. pylori* virulency-associated genes included *Cag* PAI type IV secretion system, type IV secretion system (T4SS) effectors cytotoxin-associated gene A (*cag*A), cytolethal distending toxin (*cdt*), plasticity region, capsular, cell-motility- and flagellar-associated genes, neutrophil-activating protein (*nap*), lipopolysaccharide Lewis antigens, and urease-associated genes *ureA* and *ureB* were also noted, etc. The emergence of drug-resistant microorganisms extending towards *H. pylori* strains, along with the spread of virulent factors among whole genomes, enhances the worldwide public health hazard [34]. It has recently been evidenced that the presence of antibiotic resistance and virulence factors’ combination among genomes of particular bacteria is a potential risk to community health welfare [35]. In general, virulence genes’ distribution among the *H. pylori* genomes is a multifaceted and complex phenomenon. In totality, distribution of virulence genes depends on the specific strain and virulence factor(s) involved. Currently, solving the mysteries of virulence genes’ distribution and function is a dynamic research area to develop more effective diagnostics, treatment, and prevention strategies.

## 4. Discussion

*H. pylori* strains isolated from diverse geographic regions often demonstrate phylogeographic differences. It is understood that different strains display different interaction with humans, influencing the pathological outcomes. *H. pylori* strains HPA1 and HPA2, are closely associated with *H. pylori* strains Hpfe074 and HPGA1, and these strains are known as the causative agent for many infections and increase the likelihood of ulcer disease and gastric neoplasia. Invasiveness of pathological consequences and severity of the symptoms depend upon type and expression of strain-specific VFs. Therefore, a few individuals colonized by *H. pylori* develop infection [12]. The geographic distribution and genetic diversity of *H. pylori* strains have been linked to differences in clinical outcomes. For example, certain strains prevalent in specific regions may be associated with a higher risk of certain diseases, while strains found in other regions may be less pathogenic. This suggests that the genetic makeup of *H. pylori* can impact the likelihood of developing *H. pylori*-associated diseases. Additionally, the geographic distribution of the various *H. pylori* populations across the globe appears to be associated with the disease outcomes [12]. The comparative genome analysis of Indian strains reported that number of virulent, resistant, and disease-related genes in *H. pylori* strains obtained from two diverse regions markedly fluctuates. Further, depending upon SNP-based phylogenetic analysis, region-specific *H. pylori* strains were distinguished into distinct clades. Provisionally antibiotic resistance phenotypes correlated with faster evolution rates [36].

In total, the genomic hallmarks demonstrate a disparity in the GC contents, genome size, and in gene coding sequences. Significantly, GC contents shape the amino acid sequence which relates to the protein structure classes, the translation competence, and the metabolic efficiency, determining the varied role of protein in each strain and/or species. High rates of genetic diversity are a prominent characteristic of *H. pylori.* This hyper genome variety among *H. pylori* isolated strains HPA1 and HPA2 comes from hyper mutations and frequent transfer of genetic material throughout infections induced with several *H. pylori* strains.

Geography-based differences among *H. pylori* strains, show phylogeographic variations, and the studies based on genetic variants are helpful as indicators of human migrations. Additionally, the global geographic dispersion of various *H. pylori* populations apparently related to the pathological outcomes. The diversity of *H. pylori* strains, including strains HPA1 and HPA2’s results, are possibly helpful resources to understand the diversity, epidemiology, drug resistance, and pathogenic nature following possible anti-virulent drug targets [37].

Genomically, the contrasts and categorizations of functional elements of strains HPA1 and HPA2 rely on the recognition of the natural selection pathways. These types of studies uncover a novel dimension of comparative genomics in a way that comparative genomes of five strains related with various gastric diseases comprehensively pinpoints the differences in general stages of genome structure with various drug-resistance- and virulence-related genes [38]. These findings are important for understanding the genetic basis of antibiotic resistance in these *H. pylori* strains, as well as for developing strategies to combat antibiotic resistance in clinical settings. It is also worth noting that the high similarity in some genes between strains could indicate potential transfer of antibiotic resistance genes between them, which is a concern for the development of multidrug-resistant strains. Overall, all these genes identified in this study were important, but some genes such as Iso-tRNA, *gid*B, *gyr*A, and *cme*DEF are indeed important genes associated with various drug resistance mutations. *gyr*A and *gyr*B are the two subunits of DNA gyrase enzyme. Resistance to fluoroquinolones results from a change in one or more amino acids in gyrase [39]. Using genes like *gid*B, *gyr*A may have the ability to be successful against a broad range of bacterial pathogens and may offer new options for combating antibiotic-resistant bacteria. Bacterial DNA gyrase, a type II topoisomerase, is crucial for bacterial DNA replication as well as for transcription. It is involved in introducing negative supercoils into DNA, which is necessary for various cellular processes in bacteria. Due to its essential role in bacterial DNA metabolism, DNA gyrase has been identified as a clinically relevant target to develop novel antibacterial medications [40]. Shetty et al., reported the high rates of resistance towards levofloxacin and metronidazole, whereas low resistance towards clarithromycin and *gyr*A-based new mutations responsible for levofloxacin resistance were also reported [24]. Moreover, virulent and drug-resistant strains of *H. pylori* isolated from duodenal ulcer and gastric patients have also been reported in the Indian sub-continent, such as in India and Bangladesh, previously [21,22,23,24]. These strains have been reported encoding various pathogenic genes such as *cag*A, *vac*A, *cag*A PAI, and outer membrane proteins of *hop* family in both strains. Moreover, the occurrence of various key drug-resistant genes *rdxA* and/or *frxA*, *23S rRNA* and *gyrA* were also reported [24] among sub-content strains [22].

The high prevalence of antibiotic-resistant genes in bacterial populations is a concerning and important public health issue. It underscores the need for difficult surveillance plans to monitor and track the emergence and spread of antibiotic resistance in various sources, including clinical settings, agriculture, and the environment.

Following the existence of various drug-resistant genes, strains HPA1 and HPA2 also encode various potential virulent genes. Among them, iron uptake genes were in bulk. Iron is vital to nearly every living cell. Bacterial and human cells require iron to continue their normal physiology. On the other side, mostly human diseases are correlated to pathogens’ iron utilization ability [41]. In contrast, as a nutritional defense strategy, our immune system chelates iron so that it is locked and no longer available to pathogens including bacteria [42]. Therefore, the existence of virulent genes with iron utilization capacity in *H. pylori* is of great concern because such strains can potentially obtain vital nutrients in hostile stomach environments and induce infection.

In addition to iron-uptake-related genes, multiple toxin-related genes were observed in all five *H. pylori* strains under investigation. Further, motility- and flagella-related genes were also of great importance, as bacterial pathogenicity is largely dependent on flagellar motility and chemotactic responses. A large body of evidence shows that *H. pylori* successfully colonizes gastric tissue due to flagellar activity through a thick layer of mucus. The flagella of *H. pylori* have been extensively studied, and convincing evidence is available, demonstrating the key role of these structures in the colonization of the human gastric mucosa by this fastidious pathogen. At least 50 putative proteins in *H. pylori* are forecasted to participate in the expression, secretion, and assembly of flagella [42]. More than 20 of these proteins make up the structural parts of flagella and were identified in our study.

Another group of virulent genes encoded by strains HPA1 and HPA2 were adhesions. Adhesins are bacterial cell surface proteins which facilitate the adherence process to host cell surfaces that were distributed among the *H. pylori* genomes. Adhesins are the key to successful colonization of a new host. The VFs of pathogenic bacteria can be exploited as markers and targets for antibacterial medications. Nevertheless, several *H. pylori* strains might hide under the cover of multiple VFs, and several factors are only present in a section of *H. pylori* strains. WGS offers quick and comprehensive description of virulence factors in *H. pylori,* providing valued information about the pathogenic capabilities of the *H. pylori.* It creates an opportunity to exploit more effective personalized cure and management for patients harboring *H. pylori* [43].

## 5. Conclusions

The evolutionary analysis demonstrated that there are distinct populations, or strains, of *H. pylori* linked with different geographic regions. Different populations of *H. pylori* can have varying levels of virulence and may be more prevalent in certain areas. The incidence of specific *H. pylori* populations in Pakistan can contribute to the varying rates of *H. pylori*-induced infections in several regions. The phylogeographic divergence of *H. pylori* strains from various geographical regions is evident, and research on the genetic variations can be used to identify human migratory patterns. Additionally, it appears that the clinical results are correlated with the geographic distribution of the *H. pylori* strains HPA1 and HPA2. Both strains HPA1 and HPA2 had virulence-related genes involved in adherence, motility, and most importantly iron uptake, including iron/manganese transport genes such as *horB*, *hes*, *fepG*, *entB*, *entE*, *Ferric enterobactin transport protein FepE*, *salicylate synthase Irp9,* and *enterobactin exporter* genes. These VF-related genes can be exploited for effective multipurpose medications for *H. pylori* eradication. The findings of our study provide information on the epidemiology, variety, pathogenicity, and features of drug resistance associated with possible anti-virulent medication targets.

## Figures and Tables

**Table 1:** Antibiotic resistance and sensitivity of isolated *H. pylori* strains.

Antibiotic	Resistance		Sensitive	
	No. of Strains	Percentage	No. of Strains	Percentage
Metronidazole	82	98	2	2
Ampicillin	0	0	84	100
Clarithromycin	62	74	22	26
Levofloxacin	2	2	82	98
Amoxicillin	4	5	80	95
Tetracycline	3	4	81	96

**Table 2:** Genomic features of *H. pylori* strains HPA1 and HPA2 with closely associated strains.

*H. pylori* Strain	HPA1	HPA2	HPGA1	hpfe074	ATCC 43504
Origin	Human	Human	Human	Human	Human
Accession No.	JAUDRP000000000	JAUDRQ000000000	GCA022923055.1	CP000241.1	AP017632.1
Contigs No.	24	4	1	3	2
Genome Size (Mb)	1.66	1.67	1.59	1.67	1.60
Coding Genes.	1650	1625	1596	1629	2522
G+C Content (%)	38.8	38.7	39.1	38.71	39.02
tRNA No.	33	35	36	35	35
rRNA No.	10	9	4	4	6

## Data Availability

This Whole Genome Shotgun project has been deposited at DDBJ/ENA/GenBank under the accession JAUDRP000000000 *Helicobacter pylori* HPA1 and JAUDRQ000000000 *Helicobacter pylori* HPA2. The version described in this paper is version JAUDRP000000000 and JAUDRQ000000000.

## Supplementary Materials

The following supporting information can be downloaded at: https://www.mdpi.com/article/10.3390/microorganisms11112658/s1, Figure S1: Circular genome of *H. pylori* strain HPA1 showing antibiotic resistance genes and other major genetic features. Figure S2: Circular genome of *H. pylori* strain HPA2 showing antibiotic resistance genes and other major genetic features; Table S1: Potential antibiotic resistant genes distributed in the five genome assemblies. Table S2: Occurrence of 349 VFDB annotated genes in the five *H. pylori* associate assemblies.
