# Supplementary material for: Identification, Genome Sequencing, and Characterizations of Helicobacter pylori Sourced from Pakistan
Source: Microorganisms. 2023 Oct 29;11(11):2658. doi: 10.3390/microorganisms11112658 (PMC10673187; doi:10.3390/microorganisms11112658)
Supplement: Supplementary file 1 [file microorganisms-11-02658-s001.zip › Suplmentary Materials.pdf]

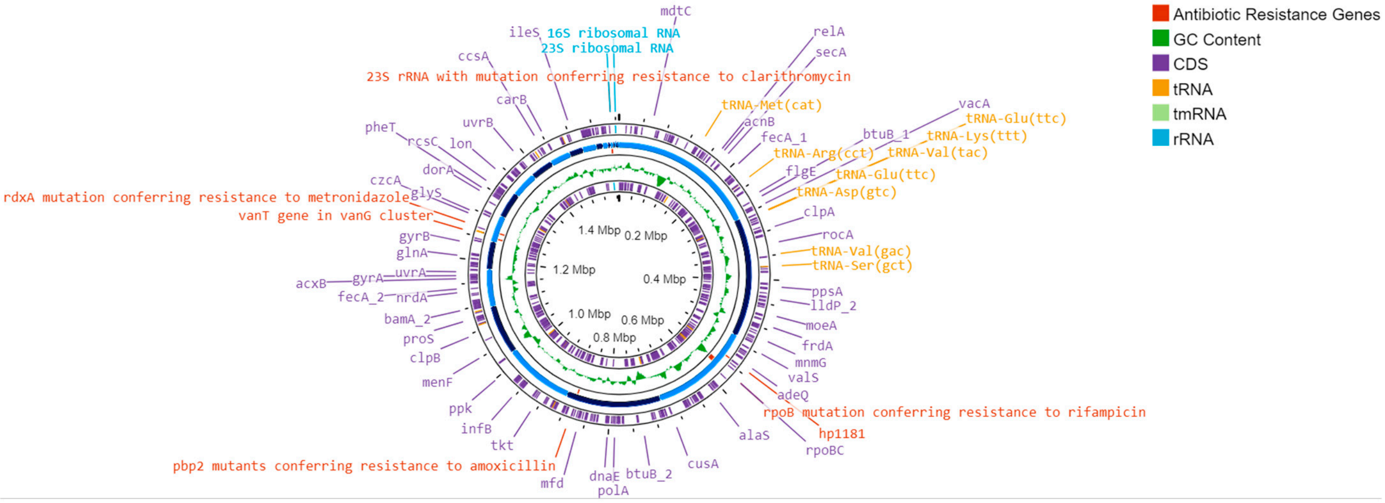

Figure S1. Circular genome of *H. pylori* strain HPA1 showing Antibiotic resistance genes and other major genetic features.

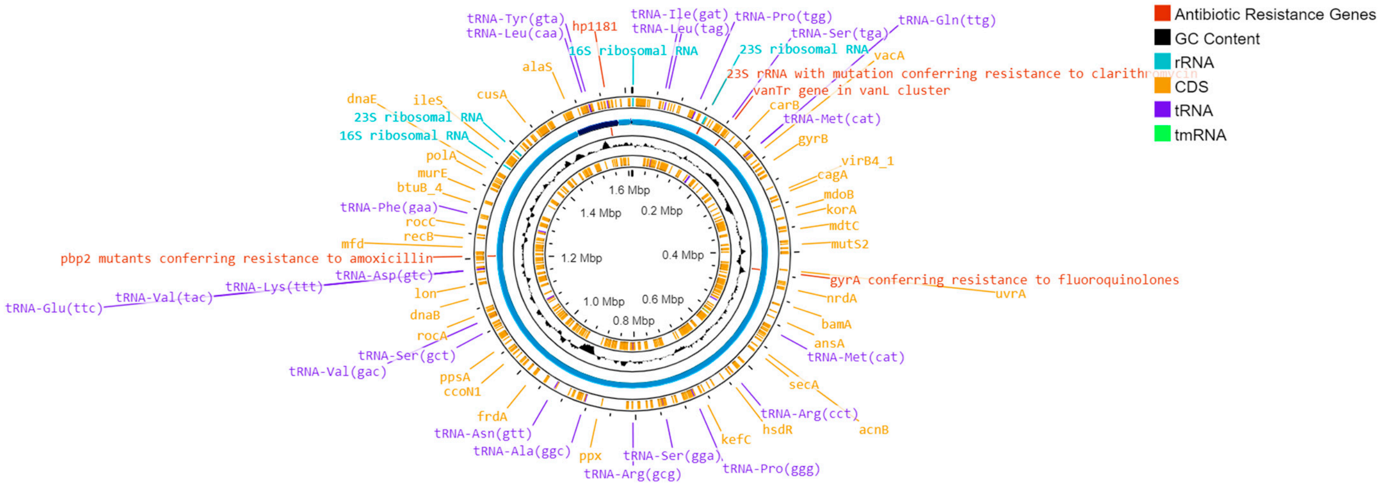

Figure S2. Circular genome of *H. pylori* strain HPA2 showing Antibiotic resistance genes and other major genetic features.

Table S1. Potential antibiotic resistant genes distributed in the five genome assemblies.

		H. pylori **HPA1**	*H. pylori* **HPA2**	*H. pylori* **HPAG1**	*H. pylori* **hpfe074**
**Name of Gene**	Product	**Best Identities**	**Best Identities**	**Best Identities**	**Best Identities**
Iso-tRNA	Isoleucyl-tRNA synthetase	98.17	98.73	91.71	98.17
gidB	16S rRNA (guanine(527)-N(7))-methyltransferase	100	100	100	100
CmeDEF	Multidrug efflux system	100	100	94.74	97.7
CmeDEF	Multidrug efflux system	99.52	99.42	99.74	98.81
S10p	SSU ribosomal protein S10p	99.52	98.39	99.95	99.09
gyrA	DNA gyrase subunit A	99.48	99.95	98.25	99.25
S12p	SSU ribosomal protein S12p	97.89	98.54	99.05	94.05
Ddl	D-alanine--D-alanine ligase	99.27	93.43	99.09	97.86
inhA, fabI	Enoyl-[acyl-carrier-protein] reductase	99.8	99.61	99.46	97.12
7a-HSDH	Enoyl-[acyl-carrier-protein] reductase	83.61	99.74	97.84	91.45
CmeDEF	Multidrug efflux system, lipoprotein	99.8	99.52	98.45	96.67
Alr	Alanine racemase	99.74	98.22	96.98	91.99
kasA	3-oxoacyl-[acyl-carrier-protein] synthase, KASII	99.9	99.63	97.78	98.15
gyrB	DNA gyrase subunit B	94.45	95.25	94.27	97.86
EF-G	Translation elongation factor G	99.07	99.43	100	99.1
PgsA	CDP-diacylglycerol--glycerol-3-phosphate 3-phosphatidyltransferase	98.8	96.52	98.56	98.45
rho	Transcription termination factor Rho	100	100	100	94.67
EF-Tu	Translation elongation factor Tu	97.9	98.63	99.78	98.58
MurA	UDP-N-acetylglucosamine 1-carboxyvinyltransferase	97.56	95.25	91.27	94.67
dxr	1-deoxy-D-xylulose 5-phosphate reductoisomerase	99.77	98.43	99.56	94.58
